# Amelioration of Abnormalities Associated with the Metabolic Syndrome by *Spinacia oleracea* (Spinach) Consumption and Aerobic Exercise in Rats

**DOI:** 10.1155/2017/2359389

**Published:** 2017-07-16

**Authors:** Vandana Panda, Kinjal Mistry, S. Sudhamani, Mukesh Nandave, Shreesh Kumar Ojha

**Affiliations:** ^1^Department of Pharmacology & Toxicology, Prin. K. M. Kundnani College of Pharmacy, Jote Joy Building, Rambhau Salgaonkar Marg, Cuffe Parade, Colaba, Mumbai 400005, India; ^2^Department of Pathology, Dr. D.Y. Patil Medical College, Nerul, Navi Mumbai, India; ^3^Department of Pharmacology, SPP School of Pharmacy & Technology Management, SVKM's NMIMS, Mumbai, India; ^4^Department of Pharmacology and Therapeutics, College of Medicine and Health Sciences, United Arab Emirates University, Al Ain, UAE

## Abstract

The present study evaluates the protective effects of an antioxidant-rich extract of *Spinacea oleracea* (NAOE) in abnormalities associated with the metabolic syndrome (MetS) in rats. HPTLC of NAOE revealed the presence of 13 total antioxidants, 14 flavonoids, and 10 phenolic acids. Rats administered with fructose (20% *w*/*v*) in drinking water for 45 days to induce abnormalities of MetS received NAOE (200 and 400 mg/kg, po), the standard drug gemfibrozil (60 mg/kg, po), aerobic exercise (AE), and a combination of NAOE 400 mg/kg and AE (NAOEAE) daily for 45 days. All treatments significantly altered the lipid profile and attenuated the fructose-elevated levels of uric acid, C-reactive protein, homocysteine, and marker enzymes (AST, LDH, and CK-MB) in serum and malondialdehyde in the heart and restored the fructose-depleted levels of glutathione and antioxidant enzymes (superoxide dismutase, catalase, glutathione peroxidase, and glutathione reductase). A significant decrease in blood glucose and insulin levels decreased insulin resistance, and improved glucose tolerance was observed in the treatment animals when compared with the fructose-fed animals. The best mitigation of MetS was shown by the NAOEAE treatment indicating that regular exercise along with adequate consumption of antioxidant-rich foods such as spinach in diet can help control MetS.

## 1. Introduction

The metabolic syndrome (MetS), also known as syndrome X, insulin resistance syndrome, or dysmetabolic syndrome, is a cluster of metabolic risk factors that come together in a single individual. The risk factors include atherogenic dyslipidemia, hypertension, hyperglycemia, abdominal obesity, insulin resistance, a proinflammatory state, and a prothrombic state [1]. MetS is the causative factor for the twin global maladies, type 2 diabetes (T2DM) and cardiovascular disease (CVD).

Dyslipidemia is an integral part of MetS which includes hypertriglyceridemia, hypercholesterolemia, low HDL, high LDL, and VLDL levels. Individuals with MetS, particularly those with abdominal obesity, exhibit a highly atherogenic lipid profile, which may account for their high risk of CVD. Central fat accumulation and the presence of insulin resistance have both been associated with dyslipidemia [2].

The MetS is known to be caused by insulin resistance, a condition whereby the body's cells are incapable of taking up glucose from the blood [3]. T2DM or noninsulin-dependent diabetes mellitus is the most common form of diabetes in which the body has adequate insulin, but the cells have become resistant to it, accounting for 90%–95% of the diabetic cases. The risk of CVD is 200% higher in diabetic population than in nondiabetic individuals. Progression of T2DM can lead to diabetic cardiomyopathy, which comprises diabetes-associated changes in the structure and function of the myocardium independent of coronary artery disease and hypertension.

Homocysteine is known to be a strong and independent marker of risk for the development of cardiovascular disease [4]. There is a graded mortality risk associated with an elevated fasting plasma total homocysteine (tHcy). MetS and T2DM being risk enhancers for CVD, hyperhomocysteinemia (HHcy) prevails in 5 to 7% of the population having MetS [5]. Declining glomerular filtration and overt diabetic nephropathy are major determinants of tHcy elevation in MetS and T2DM.

Fructose has been implicated as a contributor to nearly all of the classic manifestations of MetS. Increased fructose consumption can lead to hyperlipidemia, development of insulin resistance, inflammation, oxidative stress, obesity, and comorbidities such as hypertension and T2DM, all risk factors for cardiac dysfunction [6]. Hence, rats given free access to drinking water containing an excess of fructose exhibit all classical abnormalities associated with MetS. Fructose-enriched diet has been shown to promote oxidative stress in rodents [6]. Moreover, oxidative stress has been closely related to cardiovascular diseases linked with diabetes [7].

Recently, much attention has been focused on plant foods that may be beneficial in preventing the metabolic syndrome and possibly reducing the risk of diabetes and cardiovascular disease. Dietary patterns high in green leafy vegetables are generally found to be associated with lower prevalence of the metabolic syndrome [8].

*Spinacea oleracea* (spinach) a green leafy vegetable consumed by people across the globe is reported to possess antidiabetic, antihyperlipidemic, and antithrombotic activities by virtue of its numerous antioxidant phytoconstituents, together termed as the natural antioxidants, NAO [9]. NAO comprises flavonols, polyphenols, and vitamins (A, B1, B2, B3, B6, C, E, K, and folic acid). Eighteen flavonoids representing glucuronides and acylated di- and triglycosides of methylated and methylene dioxide derivatives of 6-oxygenated flavonols, viz., patuletin, spinacetin, and jaceidin, have been identified from spinach [10]. This wide array of antioxidant phytoconstituents present in spinach renders it a potent ROS scavenger and antioxidant.

Flavonoids found ubiquitously in most edible vegetables and fruits and constituting a major portion of micronutrients in diet have been known to possess good antidiabetic and antihyperlipidemic activities. A study on spinach has shown its flavonoids to alleviate hyperlipidemia in rats by decreasing oxidative stress [11]. Leaves of spinach have exhibited significant antihyperglycemic activity in normal and alloxan-induced hyperglycemic rats [12]. The therapeutic effect of spinach in subjects with metabolic disorders has not been reported. Aerobic exercise in any form has been proven to have positive results in diabetes, obesity, hyperlipidemia, and cardiovascular disease. The present work was therefore carried out to investigate the beneficial role of *Spinacea oleracea* along with aerobic exercise in fructose-induced abnormalities associated with MetS in rats.

## 2. Materials and Methods

### 2.1. Plant Material

Spinach was purchased from a farm on the outskirts of Mumbai, India, and authenticated at the Blatter Herbarium, St. Xavier's College, Mumbai, after matching with the existing specimen (specimen no.TK-1). The leaves were washed, shade dried, and ground to obtain a dry powder. This powder was extracted using a mixture of methanol : water (70 : 30) *v*/*v* to obtain a NAO-rich extract of *Spinacia oleracea* termed as “NAOE” and was dried and stored in a refrigerator for further use. The yield of dry NAOE from spinach leaves was 7.24% *w*/*w*.

### 2.2. Drugs and Chemicals

Epinephrine, 5,5′-dithiobis (2-nitrobenzoic acid)—(DTNB) and trichloro acetic acid (TCA) were purchased from Sigma Chemical Co., St Louis, MO, USA. Thiobarbituric acid (TBA), reduced glutathione, oxidized glutathione, and nicotinamide adenine dinucleotide phosphate (NADPH) were obtained from Himedia Laboratories, Mumbai, India. All other chemicals were obtained from local sources and were of analytical grade.

### 2.3. HPTLC Fingerprinting of NAOE for Total Antioxidants, Flavonoids, and Phenolic Acids

High performance thin layer chromatography (HPTLC) of NAOE was carried out on the CAMAG HPTLC system for the determination of natural antioxidants, flavonoids, and phenolic compounds. Total flavonoids were determined by HPTLC in our earlier studies [13]. Solutions of the reference standard rutin and NAOE (100 mg/mL in methanol) at different concentrations were applied on activated HPTLC silica gel 60 F254 plates (20 × 10 cm) and developed with an antioxidant-specific mobile phase comprising chloroform : toluene : ethanol [4 : 4 : 1 (*v*/*v*)]. The plates were then derivatized with a solution of 0.1 mM of 1,1-diphenyl-2-picrylhydrazyl in methanol, heated at 110°C, and examined in fluorescent light at 366 nm in the CAMAG visualizer. Similarly, HPTLC was performed for phenolic acids by using a solvent system comprising ethyl acetate : methyl ethyl ketone : formic acid: water (5 : 3 : 1 : 1). The plates were derivatized with 10% methanolic H_2_SO_4_ and examined in fluorescent light at 366 nm.

### 2.4. Animals

Sprague Dawley female rats (150–200 g) were acquired from Glenmark Pharmaceuticals Ltd., India. They were housed in clean polypropylene cages under standard conditions of humidity (50 ± 5%), temperature (25 ± 2°C), and light (12 h light/12 h dark cycle) and fed with a standard diet (Amrut laboratory animal feed, Pranav Agro Industries, India) and drinking water ad libitum. All animals were handled with humane care. Experimental protocols were reviewed and approved by the Institutional Animal Ethics Committee (Animal House Registration no. 25/1999/CPCSEA) and conform to the Indian National Science Academy Guidelines for the Use and Care of Experimental Animals in Research.

### 2.5. Preparation of Test Solutions and Drinking Water

Fructose solution (20% *w*/*v* in distilled water) was prepared every day and given as drinking water to animals of all test groups. NAOE was dissolved in distilled water, and the aqueous solution was used immediately for administration. Gemfibrozil was suspended in an aqueous solution of 1% carboxymethyl cellulose and administered orally.

### 2.6. Experimental Procedure

After acclimatization for 7 days in the animal quarters, rats were randomly divided into 7 groups of 6 animals each (groups I to VII) and treated in the following way.

- Group I (normal control): rats received drinking water (1 mL/kg, p.o) daily for 45 days.
- Group II (toxicant control): rats received fructose (20% *w*/*v*) in drinking water for 45 days.
- Group III (NAOE200): rats received fructose (20% *w*/*v*) in drinking water and NAOE (200 mg/kg, p.o) once daily for 45 days.
- Group IV (NAOE400): rats received fructose (20% *w*/*v*) in drinking water and NAOE (400 mg/kg, p.o) once daily for 45 days.
- Group V (standard): rats received fructose (20% *w*/*v*) in drinking water and gemfibrozil (60 mg/kg, p.o) once daily for 45 days.
- Group VI (AE): rats received fructose (20% *w*/*v*) in drinking water and aerobic exercise (swimming) for 20 min daily for 45 days.
- Group VII (NAOEAE): rats received fructose (20% *w*/*v*) in drinking water and NAOE (400 mg/kg, p.o) daily along with aerobic exercise (20 min of swimming) for 45 days.

All the animals were maintained on standard rat chow diet and drinking water ad libitum. Body weight and glucose levels were recorded once weekly. OGTT was performed on the last day of treatment, and blood glucose was measured using an automated digital glucometer (Accu-Chek Advantage, Roche, USA). At the end of the treatment period, all animals were fasted overnight, blood was withdrawn from the retro-orbital plexus, a drop was used for glucose measurement, and the remaining was allowed to clot for 30 min at room temperature. Serum was separated by centrifugation at 2500 rpm at 30°C for 15 min and used for the estimation of total cholesterol (TC), triglycerides (TGs), high-density lipoprotein (HDL), marker enzymes, viz., AST, LDH, and CK-MB, uric acid, C-reactive protein (CRP), and homocysteine (Hcy). Insulin levels were measured, and the homeostasis model assessment of insulin resistance (HOMA-IR) was calculated by the method of Matthews et al. Animals were sacrificed in the CO_2_ chamber; hearts of all animals were excised immediately, washed with ice cold saline, weighed, and divided into two parts. One part was homogenized in phosphate buffer (50 mM, pH 7.4) to prepare a 10% (*w*/*v*) solution. An aliquot was used for the determination of lipid peroxidation (LPO). The homogenates were centrifuged at 7000 ×g for 10 min at 4°C, and the supernatants were used for the assays of reduced glutathione (GSH), superoxide dismutase (SOD), catalase (CAT), glutathione peroxidase (GPX), and glutathione reductase (GR). The other part of the heart was fixed in 10% buffered formalin and used for histological studies.

Insulin resistance was determined from the formula:

(1) $$\begin{array}{c} HOMA-IR\;score=\frac{\left[serum\;glucose\;\left(mmol/L\right)\times serum\;insulin\;\left(IU/mL\right)\right]}{22:5}. \end{array}$$

LDL and VLDL were calculated as per Friedevald's equation as follows:

(2) $$\begin{array}{c} \begin{array}{l} VLDL=\frac{Total\;serum\;triglycerides}{5},\\ LDL=Total\;serum\;cholesterol-VLDL-HDL. \end{array} \end{array}$$

The atherogenic index (AI) was calculated using the following formula: AI = (TC − HDL)/HDL.

### 2.7. Oral Glucose Tolerance Test

Animals received glucose solution (1.5 g/kg) orally 30 minutes after their respective treatments. Blood glucose levels were determined at 0, 30, 90, and 120 min after glucose administration.

### 2.8. Insulin Assay

The insulin assay was carried out in serum using an ELISA kit supplied by Genxbio Health Sciences Ltd., India.

### 2.9. TC, TG, and HDL Estimation

TC, TG, and HDL levels were determined by the CHOD-PAP method, GPO-Trinder method, and the phosphotungstic acid method, respectively, using kits supplied by Erba (Mumbai, India).

### 2.10. Marker Enzyme Assays

The marker enzymes AST, LDH, and CK-MB were assayed in serum using standard kits supplied by Erba (Mumbai, India). The results were expressed as IU/L.

### 2.11. Metabolic Marker Estimation

Uric acid levels were determined by the modified Trinder's end point method using kits supplied by Erba (Mumbai, India).

### 2.12. Measurement of C-Reactive Protein

CRP was measured in serum using a standard agglutination test kit supplied by Spectrum Diagnostics, Egypt, according to the method of Pepys et al.

### 2.13. Homocysteine Assay

Hcy levels were determined in the serum of experimental animals by using an ELISA kit supplied by Cell Biolabs, Inc., USA.

### 2.14. Lipid Peroxidation

The quantification of LPO was done by determining the concentration of thiobarbituric acid reactive substances (TBARS) in the heart using the method of Ohkawa et al. [14].

### 2.15. Glutathione Estimation

GSH level was estimated in the heart homogenate using DTNB by the method of Ellman [15].

### 2.16. Antioxidant Enzyme Assays in Heart Homogenate

SOD was assayed by the method of Sun and Zigman [16]. One unit of SOD activity is defined as enzyme concentration required to inhibit the rate of auto-oxidation of epinephrine by 50% in 1 min at pH 10. CAT was estimated by the method of Clairborne [17]. GPx estimation was carried out using the method of Rotruck et al. [18]. GR activity was determined by using the method of Mohandas et al. [19].

### 2.17. Histopathological Studies

Hearts stored in 10% buffered formalin were embedded in paraffin, sections cut at 5 *μ*m, and stained with hematoxylin and eosin. These sections were then examined under a light microscope for histoarchitectural changes.

### 2.18. Statistical Analysis

The results are expressed as mean ± SEM from 6 animals in each group. Results were statistically analyzed using one-way ANOVA followed by the Bonferroni's multiple comparison test; *p* < 0.05 was considered significant. GraphPad InStat version 4.00 of Graph Pad Software Inc, San Diego, USA, was the software used for statistical analysis.

## 3. Results

### 3.1. HPTLC Fingerprinting of NAOE for Total Antioxidants, Flavonoids, and Phenolic Acids

**Figure 1:**
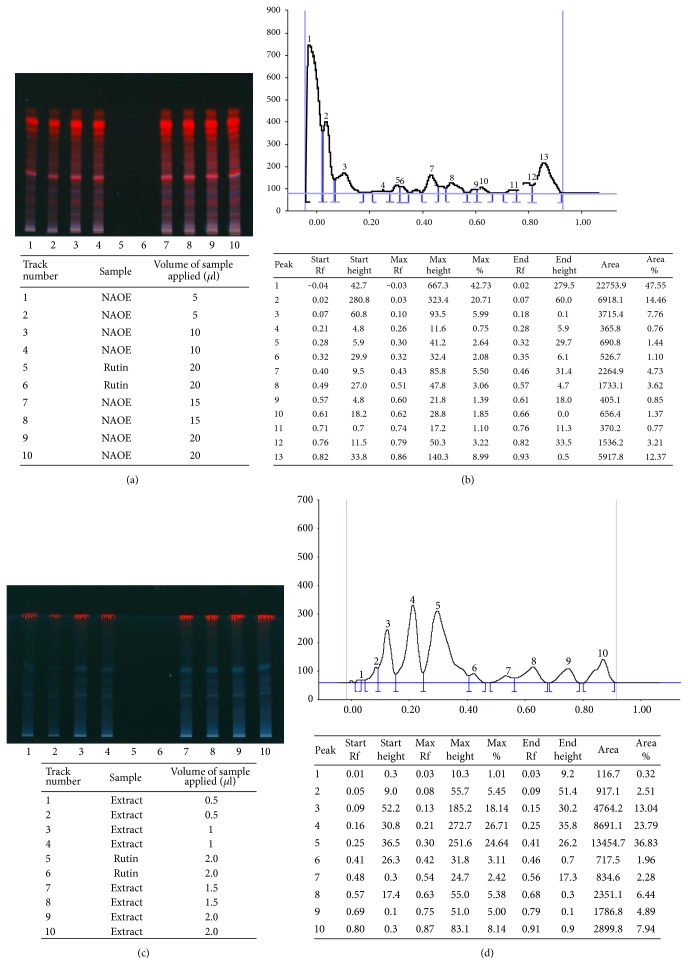
(a) HPTLC fingerprinting of NAOE for total antioxidants. (b) HPTLC chromatogram of total antioxidants of NAOE. (c) HPTLC fingerprinting of NAOE for phenolic acids. (d) HPTLC chromatogram of phenolic acids of NAOE.

HPTLC analysis at 366 nm showed clear separation of 13 antioxidants and 10 phenolic acids from NAOE (Figures 1(a), 1(b), 1(c), and 1(d)). Our earlier studies had demonstrated a good separation of 14 flavonoids [13].

### 3.2. Effect of NAOE, AE, NAOEAE, and Gemfibrozil on Body Weight

The effect of various treatments on body weight of rats recorded on the 1st, 15th, 30th, and 45th days is shown in Table 1. In the normal control animals, weight gain was observed according to the normal growth curve in the absence of fructose overload. In the toxicant (fructose control) group of rats, weight gain was significant (*p* < 0.001) on the 30th and 45th days when compared with the first day. In all treatment groups, significant reduction in weight was observed on the 30th and 45th days when compared with the day of start of the study. The NAOEAE treatment exhibited the best reduction in weight among all treatment groups against a constant stimulus of fructose overload to increase it.

### 3.3. Effect of NAOE, AE, NAOEAE, and Gemfibrozil on Blood Glucose Levels

**Figure 2:**
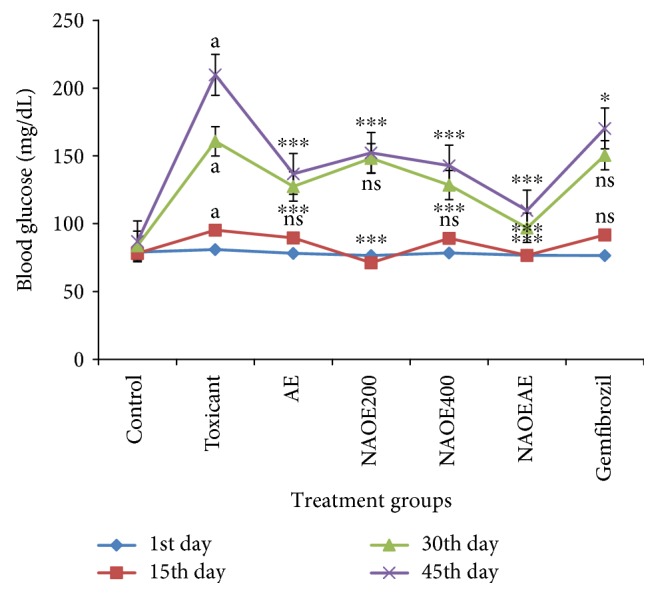
Effect of NAOE, AE, NAOEAE, and gemfibrozil on blood glucose levels in fructose-fed rats. All values are mean ± SEM; *N* = 6 in each group, one-way ANOVA followed by Bonferroni's multiple comparison tests applied for statistical analysis. *P* values: ^a^<0.001 when toxicant compared with normal control; ^∗^<0.05 when experimental groups compared with toxicant control; ^∗∗∗^<0.001 when experimental groups compared with toxicant control.

The effect of various treatments on blood glucose levels is shown in Figure 2. Administration of fructose for 45 days in drinking water to rats caused a significant (*p* < 0.001) increase in blood glucose levels when compared with the normal levels. At the end of the 45-day treatment, blood glucose levels of all treatment groups were found to be significantly lower than that of the fructose-administered group of animals. AE, NAOE, and NAOEAE treatments elicited a better effect than gemfibrozil in reducing the fructose-elevated blood glucose levels.

### 3.4. Effect of NAOE, AE, NAOEAE, and Gemfibrozil on Oral Glucose Tolerance Test (OGTT)

The effect of various treatments on OGTT is depicted in Table 2. Blood glucose levels estimated every 30 minutes under an oral glucose challenge were found to be significantly (*p* < 0.001) higher in fructose-fed rats than normal control rats indicating impaired glucose tolerance. Oral administration of NAOE, AE, NAOEAE, and gemfibrozil to fructose-fed rats significantly improved the impaired glucose tolerance. NAOE, AE, and NAOEAE treatments exhibited a better glucose-reducing effect than gemfibrozil after 120 min of glucose load.

### 3.5. Effect of NAOE, AE, NAOEAE, and Gemfibrozil on Insulin Levels and Insulin Resistance

**Figure 3:**
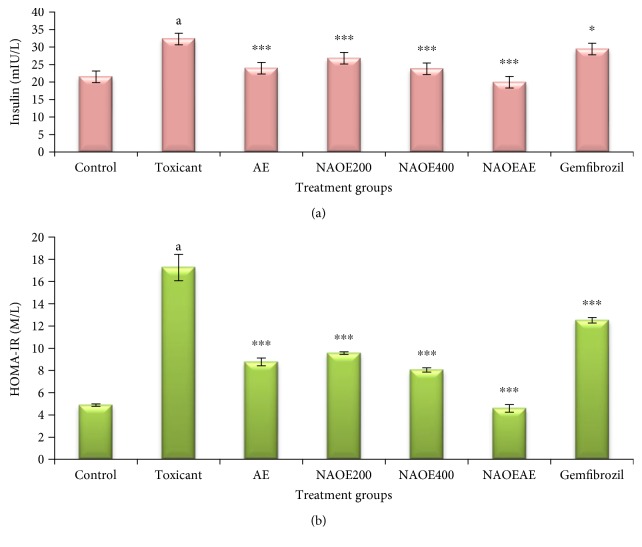
(a) Effect of NAOE, AE, NAOEAE, and gemfibrozil on insulin levels in fructose-fed rats. (b) Effect of NAOE, AE, NAOEAE, and gemfibrozil on insulin resistance (HOMA-IR) in fructose-fed rats. All values are mean ± SEM; *N* = 6 in each group, one-way ANOVA followed by Bonferroni's multiple comparison tests applied for statistical analysis. *P* values: ^a^<0.001, when toxicant control is compared with normal control; ^∗^<0.05 when experimental groups are compared with toxicant control; ^∗∗∗^<0.001, when experimental groups are compared with toxicant control.

Figures 3(a) and 3(b) represent the effect of various treatments on insulin levels and on HOMA-IR, the measure of insulin resistance. Serum insulin levels and HOMA-IR values were noted to be significantly (*p* < 0.001) elevated in fructose-fed rats when compared with the normal control rats. All treatment groups could significantly attenuate the fructose-elevated serum insulin levels and the HOMA-IR scores.

### 3.6. Effect of NAOE, AE, NAOEAE, and Gemfibrozil on Lipid Profile

**Figure 4:**
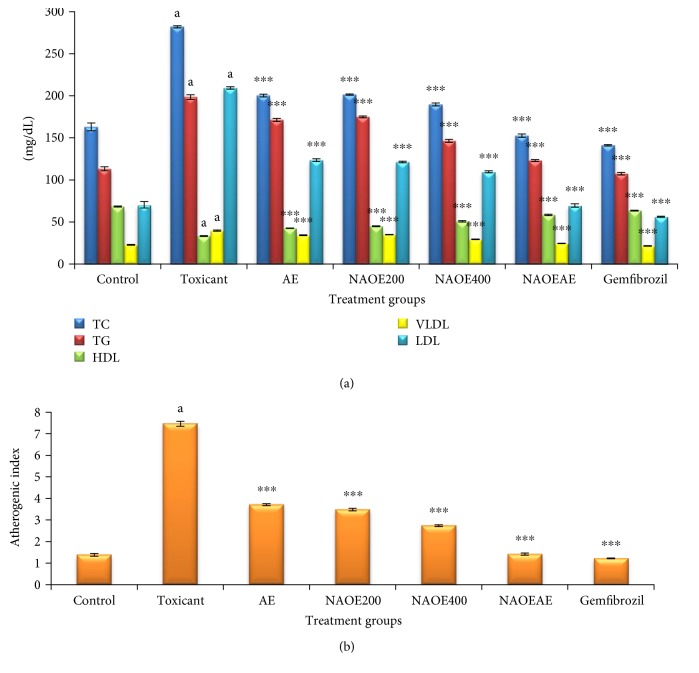
(a) Effect of NAOE, AE, NAOEAE, and gemfibrozil on lipid profile in fructose-fed rats. (b) Effect of NAOE, AE, NAOEAE, and gemfibrozil on the atherogenic index in fructose-fed rats. All values are mean ± SEM; *N* = 6 in each group, one-way ANOVA followed by Bonferroni's multiple comparison tests applied for statistical analysis. *P* values: ^a^<0.001 when toxicant control is compared with normal control; ^∗∗∗^<0.001 when experimental groups are compared with toxicant control.

The effect of gemfibrozil, NAOE, AE, and NAOEAE on lipid profile and the atherogenic index in fructose-fed rats is summarized in Figures 4(a) and 4(b), respectively. Fructose administration in drinking water to rats for 45 days significantly (*p* < 0.001) increased the levels of serum TC, TG, LDL, VLDL, and the atherogenic index and decreased the serum HDL levels in blood when compared with normal values. Gemfibrozil, NAOE, AE, and NAOEAE treatments to fructose-fed rats for 45 days significantly (*p* < 0.001) attenuated the fructose-elevated levels of TC, TG, LDL, VLDL, and the atherogenic index and elevated the fructose-depleted HDL levels.

### 3.7. Effect on NAOE, AE, NAOEAE, and Gemfibrozil on Serum Marker Enzymes AST, LDH, and CK-MB

**Figure 5:**
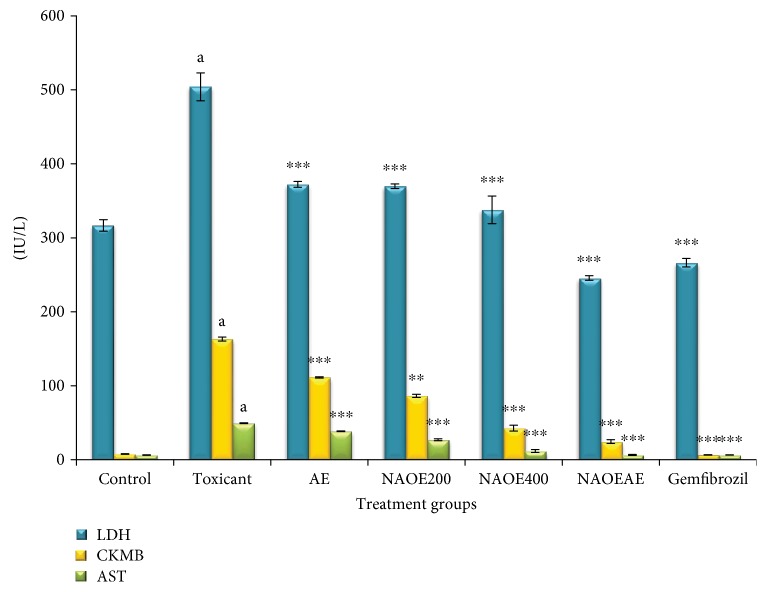
Effect of NAOE, AE, NAOEAE, and gemfibrozil on AST, LDH, and CK-MB in fructose-fed rats. All values are mean ± SEM; *N* = 6 in each group, one-way ANOVA followed by Bonferroni's multiple comparison tests applied for statistical analysis. *P* values: ^a^<0.001 when toxicant control is compared with normal control; ^∗∗∗^<0.001 when experimental groups are compared with toxicant control.

The effect of gemfibrozil, NAOE, AE, and NAOEAE on the activities of marker enzymes is demonstrated in Figure 5. Activities of the marker enzymes AST, LDH, and CK-MB were noted to be significantly (*p* < 0.001) elevated in the serum of the fructose control group of rats when compared with the normal control rats because of myocardial injury. Gemfibrozil, NAOE, AE, and NAOEAE treatments to fructose-fed rats significantly (*p* < 0.001) attenuated the fructose-elevated levels of the serum marker enzymes.

### 3.8. Effect on NAOE, AE, NAOEAE, and Gemfibrozil on Uric Acid, CRP, and Hcy

The effect of gemfibrozil, NAOE, AE, and NAOEAE on serum uric acid, CRP, and Hcy in fructose-fed rats is shown in Table 3. A significant (*p* < 0.001) increase in serum levels of CRP, uric acid, and Hcy was observed in the fructose-fed group of rats. NAOE (200 and 400 mg/kg), AE, and NAOEAE treatments brought about a significant attenuation of the fructose-elevated uric acid, Hcy, and CRP levels. However, gemfibrozil treatment could significantly attenuate the fructose-elevated CRP and Hcy levels only.

### 3.9. Effect of NAOE, AE, NAOEAE, and Gemfibrozil on LPO, GSH, and Antioxidant Enzymes

The effect of gemfibrozil, NAOE, AE, and NAOEAE on LPO, GSH, and some antioxidant enzymes is described in Table 4. MDA, the lipid peroxidation marker, was significantly (*p* < 0.001) elevated in the hearts of the fructose-fed group of rats when compared with the normal group rats. All treatments prevented significantly (*p* < 0.001) the increased formation of MDA.

Significant (*p* < 0.001) decline in cardiac GSH levels and SOD, CAT, GPx, and GR activities in the heart homogenate was observed in the fructose control group of rats when compared with the normal group of rats. NAOE, NAOEAE, and gemfibrozil treatments significantly restored the fructose-depleted cardiac GSH, SOD, CAT, GPx, and GR levels, while AE could restore significantly only the depleted GSH, SOD, CAT, and GPx levels.

### 3.10. Histopathological Studies

**Figure 6:**
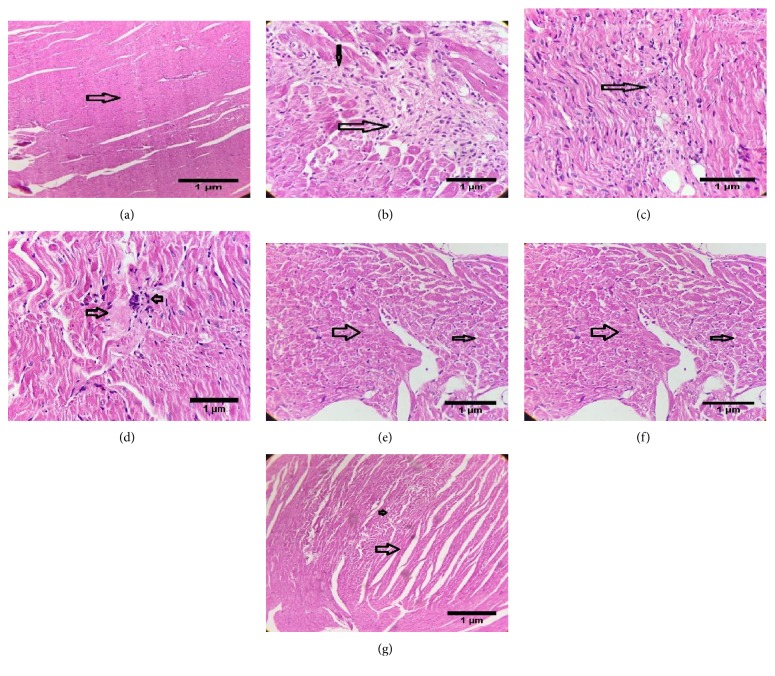
(a) Hematoxylin and eosin staining of the heart of rat of normal control group (hematoxylin-eosin, original magnification ×100). Hollow arrow indicates normal histoarchitecture of the heart. (b) Hematoxylin and eosin staining of the heart of rat of toxicant (fructose) control group (hematoxylin-eosin, original magnification ×100). Hollow arrow indicates lymphocytic infiltrate, and solid arrow indicates myocardial necrosis and severe myofibrillar loss. (c) Hematoxylin and eosin staining of the heart of rat of NAOE200 group (hematoxylin-eosin, original magnification ×100). Hollow arrow indicates splitting and swaying of myocardial fibers with moderate lymphocytic infiltrate around blood vessels. (d) Hematoxylin and eosin staining of the heart of rat of NAOE400 group (hematoxylin-eosin, original magnification ×100). The big hollow arrow indicates focal hyalinized area in myocardium, and the small arrow indicates lymphocytic infiltration. The rest of the myocardial fibers show no histomorphological abnormality. (e) Hematoxylin and eosin staining of the heart of rat of AE group (hematoxylin-eosin, original magnification ×100). Hollow arrow indicates the splitting of myocardial fibers, and solid arrow indicates minimal vacuolar degeneration. (f) Hematoxylin and eosin staining of the heart of rat of NAOEAE group (hematoxylin-eosin, original magnification ×100). Arrows indicate minimum separation of fibers. (g) Hematoxylin and eosin staining of the heart of rat with standard (gemfibrozil) group (hematoxylin-eosin, original magnification ×100). Arrows indicate minimal separation of fibers.

Histopathological studies on heart tissue of the normal control group rats (Figure 6(a)) revealed a normal histo-architecture with intact cardiac muscle fibers and blood vessels without sign of separation, edema, or any other abnormality. The heart tissue of fructose-fed rats (toxicant control, Figure 6(b)) showed myocardial necrosis, severe myofibrillar loss, and lymphocytic infiltrate. Treatment with NAOE (200 and 400 mg/kg) showed a dose-dependent decrease in splitting and swaying of myocardial fibers with moderate lymphocytic infiltrate around blood vessels. NAOE400 treatment (Figure 6(d)) showed better amelioration of damage than NAO200 (Figure 6(c)). The hearts of AE treatment group (Figure 6(e)) revealed only spitting of myocardial fibers and minimal vacuolar degeneration without necrosis or inflammatory infiltrate. Hearts of the rats of the NAOEAE treatment group (Figure 6(f)) showed maximum protection, and their histo-architecture was comparable with the hearts of the normal control group rats. Hearts of the gemfibrozil treatment group (standard, Figure 6(g)) showed myocardial histo-architecture similar to that of the normal control group except for minimal separation of fibers.

## 4. Discussion

*Spinacia oleracea* leaves extracted with a methanol-water mixture in the present study showed the presence of 14 flavonoids, 10 phenolic acids, and 13 total antioxidants in multiple HPTLC runs, confirming a high content of antioxidants in it.

The major contributing factor for progression of cardiovascular disease by fructose overload in drinking water is known to be oxidative stress [20]. Studies show that 6 weeks of fructose administration to normal rats gives rise to oxidative stress due to an imbalance between ROS production and antioxidant capacity [21]. Other studies show that chronic consumption of fructose may lead to an overpowering of endogenous antioxidants, consequently increasing ROS production and promoting oxidative stress [22].

**Figure 7:**
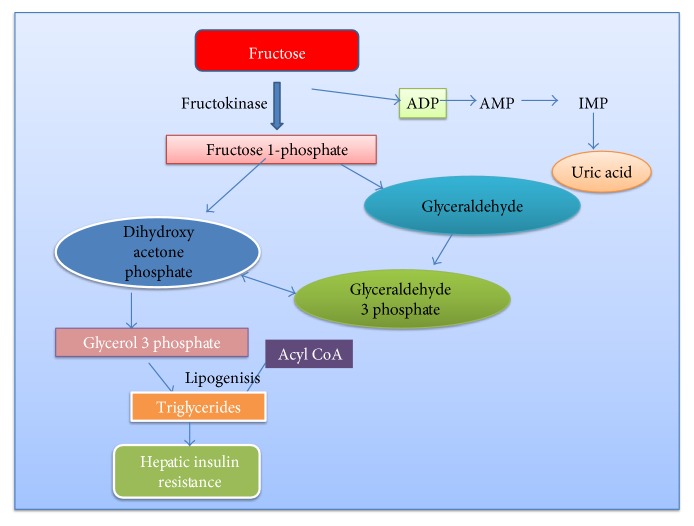
Metabolism of fructose.

Fructose intake may induce hypertriglyceridemia and lipogenesis [23]. Fructose is absorbed from the intestine via the glucose transporter GLUT 5, and it then diffuses into blood vessels through GLUT 2. Unlike glucose, fructose absorption from the intestinal lumen does not require ATP hydrolysis and is independent of sodium absorption, which results in massive fructose uptake by the liver. Fructose is converted to fructose 1-phosphate and then to two metabolites, glyceraldehyde, and dihydroxyacetone phosphate by the enzyme fructose 1-phosphate aldolase (Figure 7). In subsequent steps, the metabolites give rise to TG. The exposure of liver to large quantities of fructose leads to stimulation of lipogenesis and triglyceride accumulation and thus insulin sensitivity is reduced.

In the present study, fructose given in drinking water for 6 weeks could induce metabolic alterations such as hyperglycemia, dyslipidemia, and insulin resistance. This result indicates that a low carbohydrate diet would play a major role in ameliorating pathological conditions such as cardiovascular diseases and diabetes.

Aerobic exercise such as swimming increases oxygen consumption, number, size, and density of mitochondria and oxidative enzymes, thus increasing the rate of aerobic fat catabolism leading to energy expenditure and fat burn. Exercise also contributes to increase in the metabolic rate by improving cardiovascular capacity and suppressing proinflammatory cytokine production, in addition to increasing the release of nitric oxide and the consumption of free fatty acids (FFA), thus enhancing insulin sensitivity [24]. It has been reported that aerobic exercise, at both low and high intensity, stimulates an increase in lipoprotein lipase (LPL), improving the lipoprotein profile and enhancing the enzymatic processes involved in lipid metabolism [25]. The increase in LPL resulting from aerobic exercise can reduce TG levels and raise HDL. In this study, AE as well as NAOEAE treatments could attenuate hyperlipidemia caused by fructose consumption probably by increasing LPL activity.

Gemfibrozil is a popular fibrate from the PPAR-alpha agonists clinically used as antihyperlipidemics, more recently reported to have beneficial effects on cardiovascular function. Treatment with fibrates has reduced oxidative stress in rat hearts, reduced LDH and CK-MB in coronary effluent, decreased C-reactive protein levels and TBARS and superoxide anion generation, and consequently increased reduced glutathione levels [26]. Furthermore, fibrates have been shown to reduce oxidative stress and improve the integrity of vascular endothelium and enhance the generation and bioavailability of NO, confirming their cardioprotective potential. Hence, gemfibrozil was used as reference standard in the present study.

A major contributor to the development of insulin resistance is an overabundance of circulating FFA released from an expanded adipose tissue mass [27]. FFA reduce insulin sensitivity in muscle by inhibiting insulin-mediated glucose uptake. Increased level of circulating glucose increases pancreatic insulin secretion resulting in hyperinsulinemia. In the liver, FFA increase the production of glucose, triglycerides, and VLDL. The consequence is the reduction in glucose transformation to glycogen and increased lipid accumulation as TG.

Roden et al. demonstrated that FFA could compete with glucose for substrate oxidation, thereby increasing fat oxidation and causing insulin resistance associated with diabetes and obesity. The possible mechanism is that increased free fatty acid oxidation causes an elevation of intramitochondrial acetyl CoA and NASH/NAD+ ratios with inactivation of pyruvate dehydrogenase [28]. The inactivation of pyruvate dehydrogenase causes citrate concentration to increase, which leads to inhibition of phosphofructokinase and accumulation of glucose-6-phosphate, thus inhibiting hexokinase II, thereby decreasing glucose uptake. Thus, increase in FFA causes insulin resistance in fructose-induced rats. Oral administration of NAOE and NAOEAE to rats fed with fructose substantially prevented hyperglycemia and hyperlipidemia. NAOE treatment could alleviate insulin resistance.

Insulin resistance is closely linked to lipid metabolism as insulin resistance patients have high ectopic lipid deposition generating the toxic derivatives diacylglycerol and fatty acyl CoA. The presence of these metabolites leads to high serine phosphorylation of IRS-I (insulin receptor substrate-1) thus reducing insulin signaling and causing hyperinsulinemia [29]. Aerobic exercise is an effective intervention for improving insulin sensitivity because it increases glucose transport in insulin-sensitive tissues, especially skeletal muscle [30]. This benefit results from increases in both GLUT4 gene expression and the translocation of vesicles containing GLUT4 from the cytoplasm to the cell surface. Insulin resistance led to hyperinsulinemia due to ectopic lipid deposition in fructose-fed rats in this study. NAOEAE could significantly attenuate hyperinsulinemia than NAOE treatment probably by increasing GLUT gene expression.

Evaluation of insulin resistance (or sensitivity) and *β*-cell function is important for understanding the disease status and selection of a pharmacologic treatment. The gold standard of evaluation of insulin sensitivity is the glucose clamp test. However, the test is difficult to perform; hence, the homeostasis model assessment (HOMA-IR), first described by Matthews et al. [31], is a more commonly used method for estimating insulin sensitivity. This model is based on the theory of a feedback loop between *β* cells and the liver. The liver and beta cells function in a negative feedback loop which appears to have a predominant role in regulating both the basal plasma glucose and insulin concentrations. The HOMA-IR calculated from fasting plasma glucose level and immunoreactive insulin (IRI) is a simple method for the evaluation of insulin sensitivity and correlates with the results of glucose clamp test in subjects with mild diabetes without significant hyperglycemia.

The increased serum insulin levels and fasting glucose levels resulted in rise in HOMA-IR score in fructose-fed rats. Treatment groups showed significant reduction in HOMA-IR score indicating their ability to alleviate insulin resistance.

Fructose increases uric acid levels by the depletion of ATP and inorganic phosphate. Uric acid could be a prognostic marker for CV events in MetS. It can induce proinflammatory changes in the adipocyte that are similar to those observed in the prediabetic subjects. Uric acid inhibits endothelial function by impairing NO-induced vasodilation which is necessary for insulin to stimulate glucose uptake into tissues [32]. Thus hyperuricemia by endothelial dysfunction causes insulin resistance. Treatment groups NAOE and NAOEAE attenuated the levels of uric acid which were increased by fructose, thus alleviating hyperuricemia and insulin resistance.

MetS is associated with inflammation as evidenced by an increase in levels of the proinflammatory cytokines IL-6, resistin, TNF, and C-reactive protein (CRP). CRP is an acute phase reactant made by the liver and released into blood stream within few hours after tissue injury, the start of an infection, or other cause of inflammation. The released CRP binds to lipoproteins (LDL and VLDL), upregulating adhesion molecule expression and progression of atherosclerosis and MetS [33]. Fructose has been shown to induce oxidative stress and inflammation which may have resulted in elevated levels of CRP in the fructose-fed rats suggesting an involvement of the inflammatory process in MetS [22]. Fructose-elevated serum CRP levels were significantly attenuated by NAOE, AE, and NAOEAE treatments indicating their ability to alleviate inflammation in MetS.

**Figure 8:**
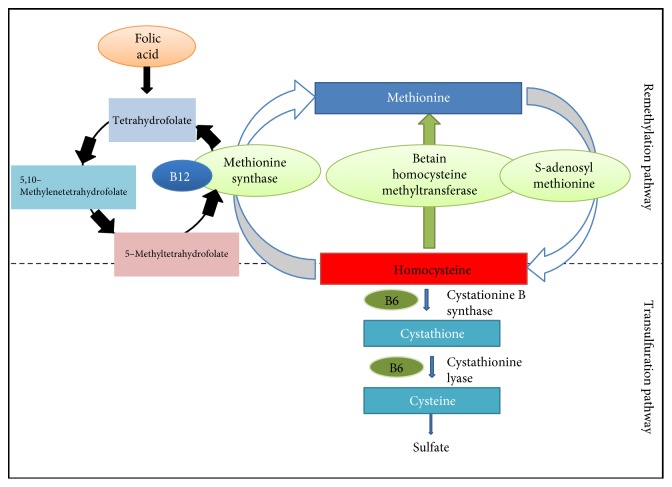
Conversion of homocysteine to methionine and cysteine.

Homocysteine is a breakdown product of the amino acid methionine in the body. Hyperhomocysteinemia is an independent and graded risk factor for the development of cardiovascular diseases. Elevated plasma homocysteine may cause or result from insulin resistance and may indicate vascular risk or be actively involved in atherogenesis [34]. Fructose-fed rats showed distinct hyperhomocysteinemia when compared with normal rats. Homocysteine should either be converted to methionine through remethylation or converted to cysteine through transsulfuration. Water soluble B vitamins have been shown to lower homocysteine levels by bringing about their remethylation or transsulfuration (Figure 8). Spinach being rich in B vitamins, NAOE treatment could significantly reduce the elevated levels of homocysteine in fructose-fed rats.

The diagnostic marker enzymes AST, LDH, and CK-MB are present plentifully in the heart. When injury to the heart occurs, these enzymes spill into blood stream. Thus, elevated levels of these enzymes released from the myocardium into blood indicate myocardial necrosis. Hyperglycemia produced by fructose overload in this model causes excessive formation of ROS which causes oxidation of the myocardial membrane leading to its damage [35]. Thus, fructose-fed rats showed increased activities of AST, LDH, and CK-MB in serum. Attenuation of the fructose-elevated marker enzyme activities seen in the NAOE and NAOEAE groups indicates significant cardioprotective activity.

High fructose administration induces oxidative stress and leads to insulin resistance and type II diabetes in rats [23]. The ROS thus formed react with PUFA of the membrane leading to peroxidation of the lipids, known as LPO. Final products of LPO are reactive aldehydes. The increased levels of MDA indicate excessive formation of free radicals by fructose and activation of the lipid peroxidative process, resulting in irreversible damage to the heart. NAOE, NAOEAE, and gemfibrozil treatment groups significantly reduced the MDA levels by preventing the formation of lipid peroxides.

Reduced glutathione is a nonenzymatic defense antioxidant present abundantly in the body. Together with GPx, GR, CAT, and SOD, it efficiently scavenges free radical species such as superoxide anions, alkoxy radicals, and H_2_O_2_. It is a substrate for the antioxidant enzymes GPx and GST and functions to protect cellular constituents from ROS and peroxides formed during metabolism. Depletion of GSH levels in fructose-fed rats may be due to its exhaustive utilization for augmenting the activities of GPx and glutathione S-transferase. Glutathione levels depleted by fructose were significantly elevated by NAOE, AE, NAOEAE, and gemfibrozil treatments. It may be understood that the increased levels of GSH could be due to its enhanced synthesis in the presence of these treatments.

SOD, CAT, and GPx constitute a mutually supportive enzyme system of the first line cellular defense against oxidative injury, decomposing O_2_^−^ and H_2_O_2_ before they combine to form the more harmful hydroxyl (OH·) radical. In the present study, SOD activity decreased significantly in the fructose-fed group of animals, may be due to an excessive formation of O_2_^−^ due to fructose-induced oxidative stress. The excessive O_2_^−^ formed lead to exhaustion of the available SOD. The activities of CAT and GPx also decreased significantly in the fructose-fed rats. SOD dismutates O_2_^−^ into H_2_O_2_which is the substrate for CAT and GPx. Due to unavailability of H_2_O_2_ as a result of SOD depletion, these H_2_O_2_ scavenging enzymes are left with no substrate to act and hence a depletion in their activity was observed. Administration of NAOE, AE, NAOEAE, and gemfibrozil to fructose-fed rats effectively prevented the decrease in SOD, CAT, and GPx activities, which may be due to scavenging of free radicals by NAOE, AE, NAOEAE, and gemfibrozil, resulting in prevention of their depletion.

GR is an antioxidant enzyme involved in reduction of GSSG (an end product formed by GPx) to GSH. In fructose-fed rats due to reduction in GPx activity, lesser GSSG was available to GR to act upon, which resulted in decreased activity of GR. Pretreatment of NAOE, AE, NAOEAE, and gemfibrozil to fructose-fed rats restored the activity of GPx, thus accelerating the conversion of GSSG to GSH by GR.

To summarize, NAOE (400 mg/kg) treatment combined with aerobic exercise for 45 days to fructose-fed rats was more effective than NAOE (200 mg/kg or 400 mg/kg) administration and AE alone. The protective activity of NAOE in MetS may be attributed to the potent antioxidant activity of its abundant NAO, especially the flavonoids. Flavonoids of NAOE stabilize the ROS by reacting with them and getting oxidized in turn to more stable less reactive radicals. Presumably, the high reactivity of OH group of flavonoids is responsible for this free radical scavenging activity [36].

Flavonoid (OH) + R^.^_→_ Flavonoid (O) + RH.

This study thus shows the way towards controlling MetS, which is a regular aerobic exercise along with adequate consumption of antioxidant-rich foods such as spinach in diet.

## Figures and Tables

**Table 1:** Effect of NAOE, AE, NAOEAE, and gemfibrozil on body weight in fructose-fed rats.

Treatment groups	Body weight (g) at different days			
	Day 1	Day 15	Day 30	Day 45
Normal control	240.33 ± 4.91	250.16 ± 4.71	259.33 ± 4.60	269.16 ± 4.09^∗^
Toxicant control	221.66 ± 5.58	251.33 ± 6.51	279.16 ± 6.88^∗∗∗^	325.83 ± 10.12^∗∗∗^
Aerobic exercise	237.50 ± 6.29	218.33 ± 6.41	203.33 ± 5.72^∗^	195.00 ± 3.65^∗∗^
NAOE200	214.16 ± 5.23	200.83 ± 2.71	189.16 ± 3.96^∗∗^	183.33 ± 4.01^∗∗∗^
NAOE400	235.00 ± 4.08	225.83 ± 3.51	208.33 ± 2.47^∗∗^	199.16 ± 1.54^∗∗∗^
NAOEAE	237.50 ± 6.29	218.33 ± 6.41	203.33 ± 5.72^∗∗∗^	193.33 ± 3.65^∗∗∗^
Gemfibrozil (60 mg/kg)	216.16 ± 5.23	200.00 ± 2.71	189.16 ± 3.96^∗^	183.33 ± 4.01^∗∗^

All values are mean ± SEM; *N* = 6 in each group, one-way ANOVA followed by Bonferroni's multiple comparison test is applied for statistical analysis. *P* values: ^∗^<0.05 when day 45, day 30, and day 15 compared with day 1 in each group for all groups; ^∗∗^<0.01 when day 45, day 30, and day 15 compared with day 1 in each group for all groups; ^∗∗∗^<0.001, when day 45, day 30, and day 15 compared with day 1 in each group for all groups.

**Table 2:** Effect of NAOE, AE, NAOEAE, and gemfibrozil on oral glucose tolerance test (OGTT) in fructose-fed rats.

Treatment groups	Blood glucose levels at different intervals (mg/dL)			
	0 min	30 min	60 min	120 min
Normal control	87.33 ± 2.37	108.51 ± 2.71	99.11 ± 2.32	86.51 ± 1.91
Toxicant control	197.21 ± 13.91^a^	227.12 ± 12.97^a^	203.71 ± 11.28^a^	189.31 ± 12.34^a^
Aerobic exercise	133.32 ± 2.56^∗∗∗^	157.31 ± 2.43^∗∗∗^	146.82 ± 2.21^∗∗∗^	133.22 ± 2.27^∗∗∗^
NAOE200	146.71 ± 3.75^∗∗∗^	177.71 ± 2.74^∗∗∗^	155.51 ± 2.95^∗∗∗^	142.31 ± 3.12^∗∗∗^
NAOE400	138.71 ± 2.86^∗∗∗^	165.51 ± 4.42^∗∗∗^	148.31 ± 3.85^∗∗∗^	130.51 ± 3.24^∗∗∗^
NAOEAE	99.67 ± 5.90^∗∗∗^	150.04 ± 7.47^∗∗∗^	129.22 ± 3.02^∗∗∗^	103.72 ± 4.44^∗∗∗^
Gemfibrozil	168.52 ± 2.29^∗^	157.51 ± 2.46^∗∗∗^	152.31 ± 2.07^∗∗∗^	155.81 ± 2.33^∗∗^

All values are mean ± SEM; *N* = 6 in each group, one-way ANOVA followed by Bonferroni's multiple comparison tests applied for statistical analysis. *P* values: ^a^<0.001 when toxicant control is compared with normal control; ^∗^<0.05 when experimental groups are compared with toxicant control; ^∗∗^<0.01 when experimental groups are compared with toxicant control; ^∗∗∗^<0.001, when experimental groups are compared with toxicant control.

**Table 3:** Effect on NAOE, AE, NAOEAE, and gemfibrozil on uric acid, CRP, and Hcy in fructose-fed rats.

Treatment groups	CRP (mg/dL)	Uric acid (mg/dL)	Homocysteine (*μ*mol/L)
Normal control	0.80 ± 0.12	0.52 ± 0.04	1.71 ± 0.04
Toxicant control	3.00 ± 0.21^a^	3.19 ± 0.05^a^	4.26 ± 0.14^a^
Aerobic exercise	1.80 ± 0.12^∗∗∗^	1.95 ± 0.05^∗∗∗^	2.32 ± 0.03^∗∗∗^
NAOE200	2.20 ± 0.12^∗^	5.30 ± 0.07^∗∗∗^	2.82 ± 0.07^∗∗∗^
NAOE400	1.60 ± 0.12^∗∗∗^	1.48 ± 0.12^∗∗∗^	1.88 ± 0.04^∗∗∗^
NAOEAE	0.90 ± 0.13^∗∗∗^	0.78 ± 0.09^∗∗∗^	2.36 ± 0.02^∗∗∗^
Gemfibrozil	1.20 ± 0.21^∗∗∗^	3.32 ± 0.05	2.64 ± 0.13^∗∗∗^

All values are mean ± SEM; *N* = 6 in each group, one-way ANOVA followed by Bonferroni's multiple comparison tests applied for statistical analysis. *P* values: ^a^<0.001 when toxicant control compared with normal control; ^∗^<0.05 when experimental groups compared with toxicant control; ^∗∗∗^<0.001 when experimental groups compared with toxicant control.

**Table 4:** Effect on NAOE, AE, NAOEAE, and gemfibrozil on LPO, GSH, SOD, CAT, GPx, and GR in fructose-fed rats.

Treatment groups	LPO (nmol MDA/min/mg protein)	GSH (*μ*mol/mg protein)	SOD (U/mg protein)	CAT (U/mg protein)	GPx (U/mg protein)	GR (U/mg protein)
Normal control	18.15 ± 0.95	3.00 ± 0.11	24.13 ± 0.26	10.69 ± 0.30	9.39 ± 0.21	242.91 ± 9.85
Toxicant control	36.18 ± 0.51^a^	0.34 ± 0.02^a^	14.81 ± 0.49^a^	4.58 ± 0.09^a^	4.96 ± 0.17^a^	87.71 ± 3.50^a^
Aerobic exercise	27.41 ± 0.85^∗∗∗^	1.54 ± 0.03^∗∗∗^	18.35 ± 0.19^∗∗∗^	6.74 ± 0.11^∗∗∗^	7.11 ± 0.14^∗∗∗^	103.61 ± 2.58
NAOE200	29.36 ± 0.75^∗∗∗^	1.97 ± 0.04^∗∗∗^	19.51 ± 0.19^∗∗∗^	7.34 ± 0.17^∗∗∗^	7.14 ± 0.13^∗∗∗^	129.12 ± 2.21^∗∗^
NAOE400	26.27 ± 0.65^∗∗∗^	2.32 ± 0.04^∗∗∗^	19.54 ± 0.47^∗∗∗^	8.81 ± 0.26^∗∗∗^	7.66 ± 0.12^∗∗∗^	150.51 ± 7.67^∗∗∗^
NAOEAE	19.15 ± 0.74^∗∗∗^	3.19 ± 0.03^∗∗∗^	22.13 ± 0.29^∗∗∗^	11.37 ± 0.23^∗∗∗^	9.34 ± 0.18^∗∗∗^	206.11 ± 8.75^∗∗∗^
Gemfibrozil (60 mg/kg)	29.36 ± 0.71^∗∗∗^	2.38 ± 0.86^∗∗∗^	21.18 ± 0.63^∗∗∗^	8.07 ± 0.12^∗∗∗^	8.33 ± 0.14^∗∗∗^	192.41 ± 5.70^∗∗∗^

All values are mean ± SEM; *N* = 6 in each group, one-way ANOVA followed by Bonferroni's multiple comparison tests applied for statistical analysis. *P* values: ^a^<0.001 when toxicant control is compared with normal control; ^∗∗^<0.01 when experimental groups are compared with toxicant control; ^∗∗∗^<0.001 when experimental groups are compared with toxicant control; 1 unit of CAT = *μ*mol H_2_O_2_ consumed/min/mg protein; 1 unit of GPX = *μ*g GSH utilized/min/mg protein; 1 unit of GR = nmol NADPH oxidized/min/mg protein.
